# Diversity of plasmids and Tn*1546*-type transposons among VanA *Enterococcus faecium* in Poland

**DOI:** 10.1007/s10096-016-2804-8

**Published:** 2016-10-17

**Authors:** E. Wardal, A. Kuch, I. Gawryszewska, D. Żabicka, W. Hryniewicz, E. Sadowy

**Affiliations:** 1 Department of Molecular Microbiology, National Medicines Institute, Chełmska 30/34, 00-725 Warsaw, Poland; 2 Department of Epidemiology and Clinical Microbiology, National Medicines Institute, Chełmska 30/34, 00-725 Warsaw, Poland

## Abstract

The objective of this study was to investigate the antimicrobial resistance, Tn*1546* transposon variability and plasmid diversity among Polish vancomycin-resistant *Enterococcus faecium* (VR*Efm*) isolates of VanA phenotype in the context of their clonal structure. Two hundred sixteen clinical VR*Efm* isolates collected between 1997 and 2010 were studied by antimicrobial susceptibility testing, MLST, MLVA and detection of IS*16*, *esp*_Efm_, *pilA*, *intA *and plasmid-specific genes by PCR. Tn*1546* structure was revealed by overlapping PCR and sequencing. Selected isolates were subjected to PFGE-S1 and Southern hybridization analyses. The vast majority of the isolates (95.8 %) belonged to lineages 17/18 (during the whole study period 1997–2010) and 78 (mostly in 2006–2010) of hospital-adapted meroclone of *E. faecium*. All isolates displayed a multi-drug resistance phenotype. Twenty-eight Tn*1546* types (including 26 novel ones) were associated with eight different ISs (IS*1216*, IS*1251*, IS*Efa4*, IS*Efa5*, IS*Efm2*, IS*Ef1*, IS*3*-like, IS*Efm1*-like). The *vanA*-determinant was typically located on plasmids, which most commonly carried rep2_pRE25_, rep17_pRUM_, rep18_pEF418_, rep1_pIP501_, ω-ε-ζ and *axe-txe* genes. VanA isolates from 1997–2005 to 2006–2010 differed in clonal composition, prevalence of gentamicin- and tetracycline-resistance and plasmidome. Our analysis revealed high complexity of Tn*1546*-type transposons and *vanA*-plasmids, and suggested that diverse genetic events, such as conjugation transfer, recombination, chromosomal integration and DNA mutations shaped the structure of these elements among Polish VREfm.

**Electronic supplementary material:**

The online version of this article (doi:10.1007/s10096-016-2804-8) contains supplementary material, which is available to authorized users.

## Introduction

In the past 20 years, vancomycin-resistant enterococci (VRE) have emerged as nosocomial pathogens worldwide. In Poland, the first VRE outbreak due to *Enterococcus faecium* (VR*Efm*) of VanA phenotype started in December 1996 in the Gdańsk Medical University [1]. The vast majority of VR*Efm* observed worldwide belongs to a specific hospital meroclone, initially described as clonal complex 17 (CC17), later divided into three distinct lineages 17, 18 and 78 based on multilocus sequence typing (MLST) analyses [2, 3]. Recently, the approach called Bayesian Analysis of Population Structure (BAPS), applied to the *E. faecium* MLST data delimited two groups within the hospital meroclone, 2–1 and 3–3, corresponding to lineages 78 and 17/18, respectively [4]. Strains belonging to the hospital meroclone are ciprofloxacin- and ampicillin-resistant, enriched in putative virulence traits, and show a distinct genetic repertoire, including cell surface protein genes (*fms)*, regulatory genes, putative pathogenicity islands, plasmids, insertion sequences (IS) and integrated phages, which promote their adaptation [5–7]. The presence of IS*16* and the *E. faecium-*specific *esp* gene (*esp*_Efm_), carried on the integrative conjugative element ICE*Efm1*, together with the *intA* integrase gene, are proven molecular markers of hospital-associated *E. faecium* [8–10].

Several glycopeptide-resistance phenotypes have been described so far, with VanA and VanB being the most common in enterococci isolated from hospital infections [11]. The *vanA* gene cluster is carried on Tn*1546*-type transposons [12], which show a significant degree of heterogeneity, associated with presence of point mutations, deletions and presence of various ISs [13, 14]. A few studies demonstrated the location of Tn*1546* on Inc18, pRUM-like, pMG1-like, and pLG1 plasmids [15, 16]; however, the knowledge of *vanA*-plasmids and their epidemiology is still far from being satisfactory and may differ significantly among countries.

In Poland, hospital VRE isolates are continuously submitted for confirmation and further analyses to the National Reference Centre for Susceptibility Testing (NRCST), located at the National Medicines Institute in Warsaw. The aim of this study was to characterize *E. faecium* VanA isolates collected by the NRCST since 1997 until the end of 2010, focusing on the Tn*1546* transposon variability and *vanA-*plasmid diversity in the context of the clonal structure of VR*Efm* isolates to provide the country-wide picture of these important hospital pathogens.

## Materials and methods

### Bacterial isolates and susceptibility testing

The study comprised 216 consecutive, non-repetitive (1 isolate per patient) VR*Efm* VanA isolates received by the NRCST from 42 hospitals in 24 cities in Poland over the period 1997–2010. Part of the isolates analyzed in this work correspond to strains partially tested in previous surveillance studies, including: 108 VanA representatives of the VR*Efm* collection from 1997 to 2005 [17] and 20 representative isolates of a *E. faecium* VanA outbreak in 2009 [18]. The majority of isolates (*n* = 137) were derived from 11 VanA outbreaks and the remaining 79 isolates were reported as single isolations. Of the 216 isolates, 211 (97.7 %) were from hospitalized patients and five (2.3 %) were from the hospital environment. Among the isolates from hospitalized patients, a total of 37 isolates (17.5 %) were from invasive infections (31 isolates from blood and 6 from other sources); 52 isolates (24.6 %) were from non-invasive infections (21 from urine, 18 from wounds, and 13 from other sources) and 122 (57.8 %) represented faecal carriage. Antimicrobial susceptibility of 88 isolates, not investigated previously, was tested by the Etest method (bioMérieux, Marcy l’Etoile, France) for daptomycin, teicoplanin and vancomycin and by a broth microdilution method [19] for the remaining compounds (Table 1). Multidrug-resistant (MDR) isolates were defined as recommended [20]. Vancomycin-resistance determinants were detected by PCR as described previously [21] with the *E. faecium* BM4147 and *E. faecalis* V583 strains as positive *vanA* and *vanB* controls, respectively.

**Table 1:** MIC values for *E. faecium* VanA isolated in Poland during the period 1997–2010

Compound/phenotype	1997–2010 N = 216	1997–2005 N = 128			2006–2010 N = 88			MIC breakpoints/ECOFF (μg/ml)	
	Number (%) non-susceptible	Number (%) non-susceptible	MIC _50_ (mg/l)	MIC_90_ (mg/l)	Number (%) non-susceptible	MIC _50_ (mg/l)	MIC_90_ (mg/l)	S ≤	R >
Vancomycin^a^	216 (100)	128 (100)	512	>512	88 (100)	>256	>256	4	4
Teicoplanin^a^	216 (100)	128 (100)	64	128	88 (100)	48	>256	2	2
Ampicillin^a^	215 (99.5)	128 (100)	>128	>256	87 (98.8)	128	>256	4	8
HLGR^a^	172 (79.6)	118 (92.2)	>1024	>1024	54 (64.4)	>1024	>1024	128	128
HLSR^a^	167 (77.3)	112 (87.5)	>1024	>2048	55 (62.5)	>1024	>2048	512	512
HLAR ^28^	147 (68.1)	110 (86)	>1024	>1024	37 (42)	>1024	>2048	-	-
Quinupristin/dalfopristin^a^	2 (0.9)	1 (0.8)	1	2	1 (1.1)	1	1.5	1	4
Linezolid^a^	1 (0.5)	0 (0)	1	4	1 (1.1)	1	2	4	4
Tigecykline^a^	0 (0)	0 (0)	0.06	0.19	0 (0)	0.06	0.25	0.25	0.5
Tetracycline^b^	135 (62.2)	89 (68.9)	64	128	46 (52.3)	8	128	4	4
Ciprofloxacin^b^	215 (99.5)	127 (99.2)	128	>256	88 (100)	128	256	4	4
Daptomycin^b^	0 (0)	0 (0)	2	3	0 (0)	2	3	4	4
Chloramphenicol^c^	53 (24.4)	30 (23.2)	8	16	23(26.1)	8	16	8	≥32^c^
MDR^28^	216 (100)	128 (100)	nc	nc	88 (100)	nc	nc	–	–

*nc* not calculated, *n* number of isolates

The results were interpreted following the European Committee on Antimicrobial Susceptibility Testing (EUCAST)-approved breakpoints [53] and the Ecological Cut-Off (ECOFF) values for compounds without defined breakpoints (http://mic.eucast.org/Eucast2/, last accessed 20th July 2015). For chloramphenicol the Clinical and Laboratory Standards Institute (CLSI) breakpoints were used [19]^a^ Interpretation according to the EUCAST clinical breakpoint value^b^ Interpretation according to the EUCAST Ecological Cut-off (ECOFF) value^c^ Interpretation according to CLSI breakpoint value

### DNA isolation and genotyping of isolates

Total DNA of isolates was extracted using Genomic DNA Prep Plus kit (A&A Biotechnology, Gdansk, Poland). Multilocus VNTR analysis (MLVA), MLST, and detection of 19 *rep* families and the unique *rep*_pMG1_ gene were performed as described [22–24]. Sequence types (STs) were grouped to CCs by the comparative eBURST analysis performed against the whole *E. faecium* MLST database. PCR detection of IS*16, esp*_Efm,_ *fms21 (pilA), rep*_pLG1,_ plasmid addiction systems, relaxase genes, and *intA*_ICE*Efm1*_ was performed as described (Supplementary Table 1 and references therein). DNA of enterococcal isolates from our laboratory collection [17, 18, 25] served as positive controls.

### Plasmid profiling, hybridization analyses, Tn*1546* typing and statistical analysis

**Fig. 1:**
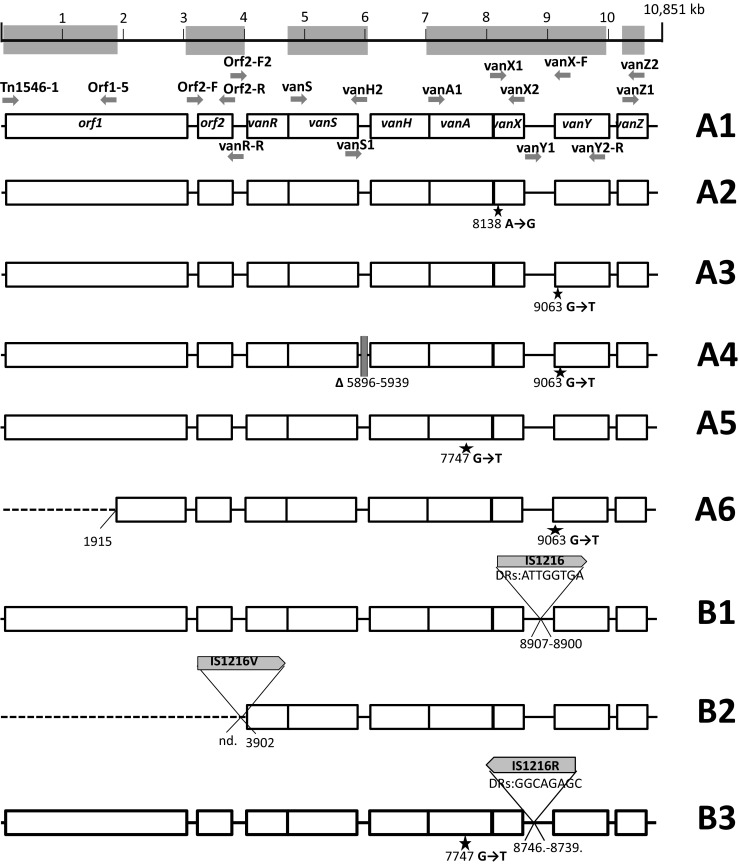

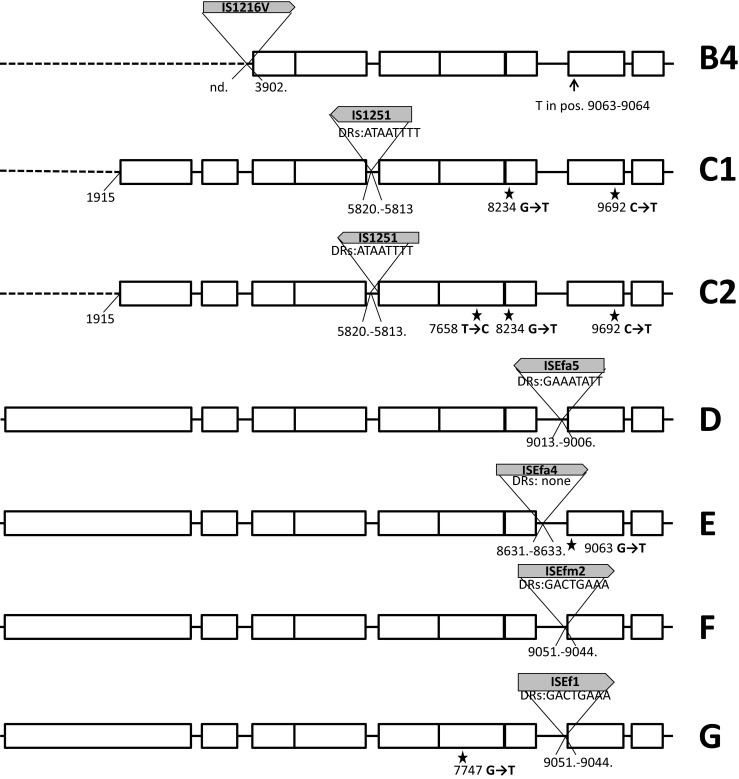

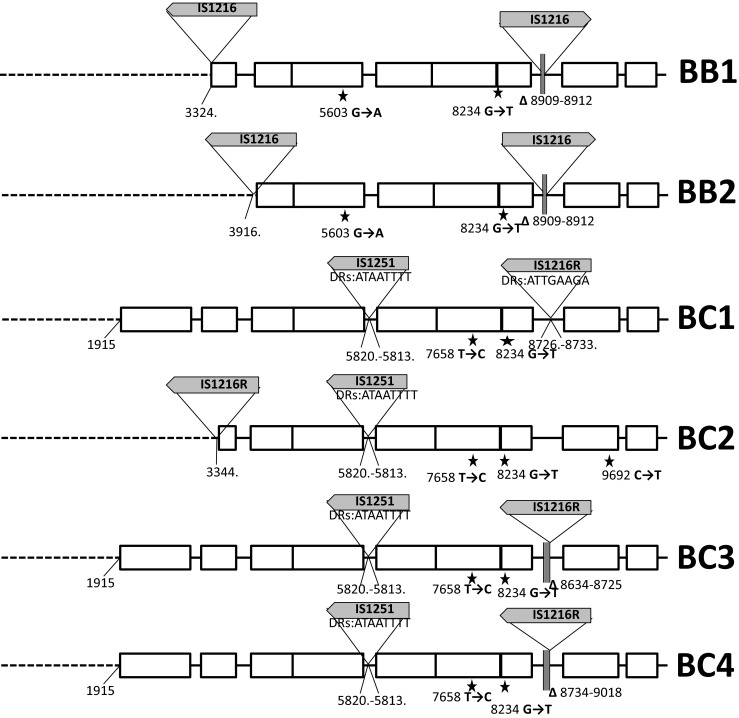

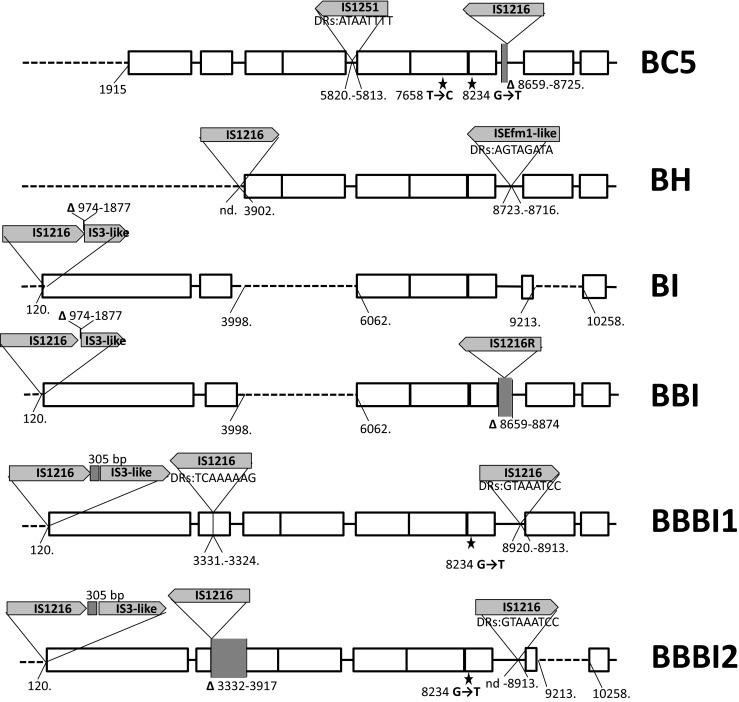
Diversity of Tn*1546* transposon types among *E. faecium* VanA isolates. Position of primers used in PCR mapping and sequencing indicated by *arrows* with primer names; *open rectangles*, transposon genes; *stars*, positions of point mutations; analyzed areas of the transposon shadowed; *dashed lines*, deletions in the *left arm* of the transposon; *filled rectangles*, deletions within the transposon; *vertical arrow*, *triangles with arrows*, the IS positions; single-nucleotide insertion in *vanY*

DNA in agarose plugs was obtained as described [21], treated with S1 nuclease (Takara Bio, Japan) and separated by PFGE with Lambda Ladder PFG marker (New England Biolabs, Beverly, MA) [26] followed by blotting onto the Hybond membrane (GE Healthcare, Buckinghamshire, UK) by capillary transfer. Hybridization was carried out using the Amersham ECL Random-Prime Labelling and Detection System (GE Healthcare, Buckinghamshire, UK). Tn*1546* transposon was investigated by PCR mapping and sequencing (Supplementary Table 1 and references therein) of selected regions encompassing 7571 bp out of 10851 bp (i.e., ∼70 % of the transposon, Fig. 1). The Tn*1546* sequence of *E. faecium* BM4147 (GenBank acc. no.: M97297) [12] was used as a reference. The nomenclature of Tn*1546*-type transposons in the present study was based on the following alphanumeric code: the ‘A’ types (A1-A6) referred to transposon variants of the wild type (*wt*) Tn*1546* structure (A1) not interrupted by insertion sequences; the ‘B’ types contained 1–3 copies of IS*1216* (B, BB, BBB types); the C, D, E, F, G, H and I types carried IS*1251*, IS*Efa5*, IS*Efa4*, IS*Efm2*, IS*Ef1*, IS*Efm1*-like and IS*3*-like elements, respectively. Transposons with more than one IS type were described by a two-, three- or four-letter code (e.g., ‘BC’ with both IS*1216* and IS*1251*). The Arabic numerals indicated differences in the presence of particular point mutations as well as the orientation of ISs and the localization of their insertion sites (e.g., B1-B4). The novel IS*Efm1*-like sequence was submitted to GenBank (KT719407). Chi-square test was used to assess the differences of distributions, with *p* ≤ 0.05 considered significant.

## Results

### Susceptibility to antimicrobial agents

All isolates were resistant to vancomycin and teicoplanin (Table 1) and carried *vanA*. Resistance to ampicillin, ciprofloxacin, tetracycline, chloramphenicol, gentamicin and streptomycin (high level) was prevalent or highly prevalent and all isolates showed the MDR phenotype. A significant decline in the prevalence of both tetracycline-resistance (from 68.9 to 52.3 %, *p* = 0.01) and high-level gentamicin resistance (from 92.1 to 64.4 %, *p* < 0.0001) was found between the 1995–2005 and 2006–2010 periods. A single isolate was resistant to linezolid and two isolates to quinupristin/dalfopristin. All isolates were susceptible to tigecycline and daptomycin.

### MLVA, MLST, IS*16* and virulence markers detection

MLVA was performed for 196 isolates and these results were analysed together with data obtained earlier for 20 isolates from the 2009 outbreak [18]. Among 216 isolates, 37 different MLVA types (MTs) and three incomplete profiles (due to lack of VNTR7 amplification) were observed, that included 207 and nine isolates, respectively (Supplementary Table 2). MT1, MT159, MT25 and MT13 were most prevalent, with 36, 34, 26 and 20 isolates, respectively. All MT159 isolates except one were isolated in 2006–2010 (*p* ≤ 0.0001), in contrast to isolates of MT25, which all except one were isolated in 1997–2005 (*p* = 0.0001). The MT1 isolates showed a similar frequency over the whole study period (13.2 % vs 21.6 %, *p* = 0.07). In the present study, STs and the presence of IS*16*, *esp*_Efm_, *intA* and *pilA* were determined for 88 isolates not encompassed by the previous studies (i.e., 68 isolates from 2006 to 2010 and 20 isolates from 2001 to 2002), and these results were analysed with the data obtained previously for the remaining 128 isolates. Altogether, 40 different STs were found, with 18 STs (45.0 %) represented by single isolates. The vast majority of the STs, i.e. 36 STs representing 207 isolates (95.8 %), belonged to the hospital-adapted meroclone of *E. faecium;* lineage 17/18 included 31 STs with 165 isolates and was present during the whole period 1997–2010, while lineage 78 included five STs characteristic for 42 isolates, occurring mostly in 2006–2010 (*p* ≤ 0.0001). IS*16* was present in 207 (95.8 %) isolates and 186 (86.1 %) isolates harboured the *esp*_Efm_ gene. The integrase gene *intA* and the *pilA* gene were found in 181 (83.8 %) and 214 (99.1 %) isolates, respectively.

### Structural diversity of Tn*1546* transposons

The structure of Tn*1546*-type transposons was determined for 187 isolates while for 20 isolates the structure of the transposon (representing types A1 and G, Fig. 1) had been published earlier [18]. In the case of nine isolates the structure of the transposon could not be determined in spite of repeated attempts due to lack of PCR products for some parts of the transposon, which might have been caused by sequence polymorphism(s) within PCR primers annealing sites, and discrepancies of sequencing results in certain regions, likely associated with the presence of more than one transposon in a single isolate. Twenty-eight transposon types, including 26 new ones, were discerned in the analyzed group (Fig. 1). The most predominant types, including C1 (40 isolates), B2 (*n* = 38), A3 (*n* = 36), G (*n* = 25), E (*n* = 14), A1 (*n* = 13) and D (*n* = 7), were associated with several STs and typically showed a multicenter distribution. Eight different ISs were detected within Tn*1546*, including IS*1216*, IS*1251*, IS*Efa4*, IS*Efa5*, IS*Efm2*, IS*Ef1*, IS*3*-like and IS*Efm1*-like. The most common IS*1216* was present in all 16 B-type transposons, both in the direct and reversed orientations, with five different types of 8-bp direct repeats. These B-type transposons were found in Gdańsk and Warsaw, as well as in 14 other cities. IS*1251* was associated with seven Tn*1546* types (C1-C2, BC1-BC5) and present in 53 isolates (25.6 %), which mainly originated from Kraków and Warsaw. In these isolates, IS*1251* was always inserted in the *vanS-vanH* intergenic region of the transposon at the position 5813. The D, E, F and G types of transposon were characterized by the presence of IS*Efa5*, IS*Efa4*, IS*Efm2* and IS*Ef1* in the *vanX-vanY* intergenic region, respectively. These types were generally limited to one or two centres, with the exception of the G type, which apart from the outbreak in two Warsaw hospitals [18] occurred in seven other centres. An insertion of an IS in 98 % identical to IS*Efm1* (GenBank no. AF138282) in the *vanX-vanY* intergenic region resulted in the BH-type of transposon.

The variability of Tn*1546* was additionally associated with the presence of deletions, insertions and point mutations. In the A4 type, a 44 nt deletion in the *vanS*-*vanH* intergenic region (nt 5896–5939) was observed. Other deletions, located in *orf2*, *vanX* and the *vanX-vanY* intergenic region coincided with the presence of ISs (six types: BB1, BB2, BC4, BC5, BBI, BBBI2). Seven different point mutations were detected, including four known previously (T7658C, G7747T, G8234T, C9692T) [18, 27, 28] and three new ones (G5603A, A8138G and G9063T). The G5603A mutation resulted in the A80T change in VanS and the G9063T mutation in the L4F change in VanY. The B4 type, found in two independent isolates, demonstrated the presence of a novel single-nucleotide T insertion between nt 9063–9064 within the *vanY* gene, resulting in translational frameshift and a truncated VanY. Nevertheless, these two isolates showed high MIC values for vancomycin (>512 mg/L in both cases) and teicoplanin (64 and >128 mg/L).

### Plasmid gene content among VR*Efm* and diversity of *vanA*-plasmids

**Fig. 2:**
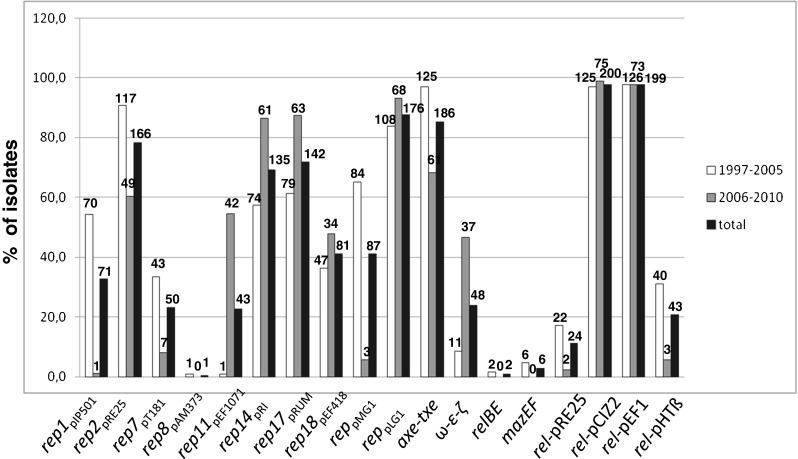
Plasmid-associated gene distribution among Polish VR*Efm* VanA. Number of isolates with a particular gene given above the graph bars

PCR-based typing of plasmid replication initiator genes (*rep*) was performed for 196 isolates and these results were combined with the data for 20 isolates, published previously [18]. Altogether, ten *rep-*types were observed among VR*Efm*-VanA (Fig. 2). Isolates carried from one up to seven different *rep* genes, with 4.7 *rep* genes per isolate on average. Isolates positive for *rep1*_pIP501_, *rep7*_pT181_ and *rep*_pMG1_ appeared mainly in the period 1997–2005, while *rep11*_pEF1071_ gene was typical for isolates obtained in 2006–2010. The plasmid stabilization systems *axe-txe* and *rep17*_pRUM_ were present in the majority of isolates. Another system, ω*-ε-ζ*, was also quite common, predominantly among *rep2*_pRE25_-carrying strains, and occurred mainly in the period of 2006–2010. Two additional systems, *mazEF* and *relBE*, were observed only in 2003 for six and two isolates, respectively. The *rel*_pCIZ2_ and *rel*_pEF1_ relaxase genes were prevalent, and additionally *rel*_pHTß_ and *rel*_pRE25_ were detected. The majority of *rel*_pRE25_-positive isolates (*n* = 22, 91.7 %) were also *rep2*_pRE25_-positive, however, most of 169 *rep2*_pRE25_-positive isolates lacked this relaxase. Isolates carrying *rel*_pHTß_ dominated in 1997–2005 (40 out of 45 *rel*_pHTß_-positive isolates) and the majority of *rel*_pHTß_ -positive isolates also harboured *rep*_pMG1_ (*n* = 43, 95.5 %).

Fifty-two isolates, obtained from 24 medical centres over the whole study period and representing 26 different STs and 21 Tn*1546* types were selected for PFGE of S1-digested DNA and hybridization analyses. Additionally, the results obtained previously for three isolates from the 2009 outbreak [18] were included for comparative purposes. Investigated isolates showed the presence from one up to 11 plasmid bands per isolate in PFGE-S1 analyses. Subsequent hybridization with the *vanA* probe revealed the presence of 86 *vanA-*plasmids with up to four such plasmids in an isolate, and two cases of chromosomal localization of *vanA* (Table 2). Further hybridization studies showed the co-localization of *vanA* determinants with all six tested *rep* types, including *rep2*_pRE25_, *rep17*_pRUM_, *rep18*_pEF418_, *rep1*_pIP501_, *rep*_pLG1_ and *rep*_pMG1_ that accounted for 40.7 % (*n* = 35), 40.7 % (*n* = 35), 24.4 % (*n* = 21), 19.8 % (*n* = 17), 5.8 % (*n* = 5) and 1.2 % (*n* = 1) of *vanA*-plasmids, respectively. The *vanA*-plasmids with *rep1*_pIP501_ were limited to isolates from 1997 to 2005, circulating in two hospitals in Poznań. These plasmids differed by size (from ca. 30 to ca. 265 kb) and presence of other *rep* and toxin-antitoxin genes, and carried four different types of Tn*1546*, with A3 being predominant (8 out of 13 isolates harbouring *vanA-*plasmids with *rep1*_pIP501_). The *vanA-*plasmids with *rep2*_pRE25_ *, rep17*_pRUM_ and *rep18*_pEF418_ genes showed a multicentre distribution and occurred during the whole study period. In total, 37 (43 %) *vanA-*plasmids were associated with more than a single *rep* type and 21 *vanA-*plasmids (24.4 %), present in 11 isolates, did not hybridize with any of the tested *rep* genes. With a single exception, these latter plasmids were obtained during 2006–2010 (*p* = 0.001). Five of the isolates with these unknown replicons concomitantly carried three *vanA-*plasmids, ca. 30, 160 and 380 kb in size, which did not hybridize with any probes of toxin-antitoxin and relaxase genes tested. All these isolates carried B2 transposons, but belonged to diverse STs and MTs, and originated from four different medical centres over 2006–2010. Two toxin-antitoxin systems, ω*-ε-ζ* and *axe-txe* were commonly carried by *vanA-*plasmids (35 and 32 plasmids, respectively). The ω*-ε-ζ* system was characteristic for *rep2*_pRE25_ plasmids and *axe-txe* for *rep17*_pRUM_ plasmids (71.4 and 62.9 % of the respective *vanA-*plasmids). The gene specifying pEF1-relaxase was located on 11 *vanA-*plasmids (12.8 %), with various *rep* types. Some of the presumed genetic events, that could be inferred on the basis of these analyses, include examples of transposon evolution within an enterococcal strain, Tn*1546* transposition among plasmids, conjugative transfer of plasmids, and their changes such as recombination or chromosomal integration as proposed in Table 2.

**Table 2:** Characteristics of the VanA plasmidome of 55 selected VR*Efm* isolates

Strain ID/year of isolation	Code of medical centre^a^	Tn*1546* type	MLST type (lineage)	Number of VanA plasmid bands	Hypothetical genetic event ^b^	VanA plasmid replicon types and stabilization systems (approximate size in kb)^c^		
1639/1997	*Gd-a*	BBBI1	407(17/18)	1		rep17 TA1 (45)		
1641/1997	*Gd-a*	BBBI2	408(17/18)	1	Chromosomal integration of 50-kb plasmid	rep17 rep18 TA1 (50)	rep2 rep17 rep18 TA1 (chr)	
3132/1998	*Gd-p*	A1	18(17/18)	2	Transfer of 40-kb plasmid between ST18 and ST411 strains, followed by plasmid recombination or transposition of A1	rep2 rep17 TA1 (35)	rep2 TA2 (40)	
3136/1998	*Gd-p*	A1	411 (singleton)	2			rep2 TA2 (40)	rep2 TA2 (270)
7952/1999	*Gd-p*	nt	381(17/18)	3		rep2 rep17 TA1 (35)	rep17 TA1 (170)	rep17 TA1 (320)
2509/2000	*Po-1*	A3	386(17/18)	2	Chromosomal integration of 40-kb plasmid	rep2 (30)	rep2 TA1 TA2 (40)	rep1 rep2 TA1 TA2 (chr)
2524/2000	*Po-1*	A3	382(17/18)	2		rep17 TA2 (40)	rep1 TA2 relpEF1(140)	
2712/2000	*Po-1*	A3	385(17/18)	3	Transposition of A3 or plasmid recombination in ST385 strain	TA2 (40)	rep1 TA2 (140)	rep1 rep_pLG1_ TA2 relpEF1 (265)
1409/2002	*Po-4*	A3	385(17/18)	1		rep2 rep_pMG1_ TA1 TA2 (250)		
2506/2000	*Po-1*	A4	385(17/18)	2	Derivative of A3 in ST385 strain, with concomitant change of plasmid backbone	rep2 rep18 TA2 (<30)	rep1 rep18 TA2 (265)	
291/2002	*Po-2*	A3	117(17/18)	1		rep1 rep17 TA1 TA2 (145)		
1714/2003	*Po-2*	A3	117(17/18)	1	Clonal spread of ST117 with 265-kb plasmid, followed by transposition of A3 among plasmids or plasmid recombination	rep1 (265)		
710/2004	*Po-2*	A3	117(17/18)	4		rep1 (265)	rep1 rep2 rep18 TA1 TA2 (255,310,360)	
914/2002	*Po-2*	A3	17(17/18)	1		rep1 rep17 TA1 (155)		
2039/2003	*Po-2*	A3	410(17/18)	1		rep1 (145)		
3010/2003	*Po-2*	A3	192(78)	1		rep1 rep2 rep18 (145)		
655/2007	*Po-4*	A6	549(78)	1		rep2 TA1 TA2 (40)		
3003/2003	*Po-2*	E	117(17/18)	1	Transposition of E or plasmid recombination in ST117 strain	rep1 rep2 rep18 rep_pLG1_ TA1 (165)		
2127/2004	*Po-2*	E	117(17/18)	1		rep1 rep_pLG1_ (165)		
2512/2000	*Po-1*	D	17(17/18)	1		rep1 TA2 (30)		
1156/2002	*Po-2*	D	382(17/18)	1		rep1 rep2 TA2 (50)		
714/2003	*Kr-1*	C1	117(17/18)	1		rep2 rep18 rep17 rep_pLG1_ TA1 TA2 (450)		
756/2003	*Kr-1*	C1	17(17/18)	1		rep17 rep18 TA1 TA2 relpEF1 (70)		
1679/2003	*Kr-1*	C1	18(17/18)	1	Clonal spread of ST18 strain and concomitant change of plasmid size by a presumable deletion (loss of rel_pEF1_)	rep2 rep18 TA1 TA2 relpEF1 (70)		
3779/2004	*Kr-4*	C1	18(17/18)	1		rep2 rep18 TA1 TA2 (65)		
4002/2005	*Kr-3*	C1	387(17/18)	1	Plasmid transfer and ∼15-kb deletion	rep2 rep18 TA1 TA2 relpEF1 (55)		
1332/2003	*Kr-1*	C1	132(17/18)	1	Clonal spread of ST132 strain with 45-kb plasmid harbouring C1 transposon	rep17 TA1 (45)		
1336/2003	*Kr-1*	C1	132(17/18)	1		rep17 TA1 (45)		
2981/2003	*Mi*	nt^d^	132(17/18)	1	Evolution of C1 transposon within the same ST132 strain and 45-kb plasmid backbone	rep17 TA1 (45)		
2216/2005	*Kr-1*	C1	388(17/18)	1		rep2 rep17 rep18 TA1 TA2 relpEF1 (75)		
84/2010	*Gdy*	C2	17(17/18)	1		rep2 rep17 TA1 TA2 (50)		
3552/2009^e^	*Wa-10*	A1	18(17/18)	2	Ancestor for B2 transposon, associated with plasmids of unknown *rep*-type(s)	(<30)	(170)	
3240/2006	*Po-5*	B2	17(17/18)	3	Concomitant transfer of three ∼30-, 160- and 380-kb plasmids with unknown *rep*-type(s), carrying B2 transposon, into diverse clonal backgrounds	(30)	(160)	(380)
1930/2007	*Wa-1*	B2	64(17/18)	3		(30)	(160)	(380)
4285/2008	*Wa-1*	B2	192(78)	3		(30)	(160)	(380)
5151/2008	*Osw*	B2	18(17/18)	3		(30)	(160)	(380)
1767/2010	*Wa-3*	B2	780(17/18)	3		(<30)	(160)	(380)
5009/2009	*Wa-2*	B2	230(78)	3	Transfer of 160-kb plasmid of unknown *rep*-type, followed by transposition of B2 among plasmids or plasmid recombination	rep17 TA1 (<30), rep17 (45)	(160)	
3238/2006	*Sk*	B2	279(17/18)	2	Transfer of 30-kb plasmid of unknown *rep*-type, followed by transposition of B2 among plasmids or plasmid recombination	(30)	rep18 rep_pLG1_ (180)	
2546/2008	*Gr*	BH	202(17/18)	3	BH derivative of B2 transposon on ∼370-kb plasmid of unknown *rep*-type, followed by transposition of BH among plasmids or plasmid recombination	rep17 rep18 TA1 (30)	rep17 rep18 TA1 (155)	(370)
8744/2010	*Wa-2*	B2	561(17/18)	1		rep2 rep17 TA2 relpEF1(35)		
484/2010	*Ke*	B2	17(17/18)	3	Transfer of <30-kb plasmid among strains of ST877 and ST17	rep2 rep17 rep18 TA1 (<30)	rep2 (160)	rep2 rep17 rep18 TA1 (320)
9363/2010	*Sw*	B2	877(17/18)	1		rep2 rep17 rep18 TA1 (<30)		
3856/2005	*Wa-1*	B4	78(78)	1		rep2 TA2 (50)		
991/2009^e^	*Wa-4*	G	18(17/18)	1	Recombination events or transposition of G among *rep17* plasmids.	rep17 (100)		
3554/2009^e^	*Wa-10*	G	192(78)	1		rep17 (50)		
2944/2009	*Ko*	G	18(17/18)	1		rep2 rep17 TA2 (35)		
3392/2009	*Ost*	G	78(78)	1		rep17 relpEF1 (45)		
726/2010	*In*	G	17(17/18)	1		rep17 relpEF1 (100)		
3322/2007	*Wa-2*	BC1	412(78)	1	Evolution of BC transposons within the same ST412 strain in the ∼35-kb plasmid backbone	rep2 rep17 TA2 (35)		
107/2010	*Wa-2*	BC5	412(78)	1		rep2 rep17 TA2 (35)		
3948/2010	*Wa-2*	BC4	412(78)	1		rep2 rep17 TA2 (35)		
1901/2005	*Lo*	F	279(17/18)	1		rep17 rep18 TA2 (50)		
8034/2010	*Kr-5*	B3	341(78)	2		rep2 rep17 TA1 TA2 relpEF1(50)	rep2 rep17 TA2 relpEF1(65)	
8628/2010	*Ka*	BBI	202(17/18)	1		(115)		

*nt* non-typeable

^a^ *By* Bydgoszcz, *Gd-a* Gdańsk, adult hemathology ward; *Gd-p* Gdańsk, paediatric haematology ward; *Gdy* Gdynia, *Gr* Grodzisk Mazowiecki, *In* Inowrocław, *Ka* Katowice, *Ke* Kętrzyn; *Ko* Konin, *Kos* Kościerzyna, *Kr* Kraków, *Lo* Łódź, *Mi* Mielec, *Op* Opole, *Os* Ostrów Mazowiecki, *Osw* Ostrów Wielkopolski, *Ost* Ostrzeszów, *Ot* Otwock, *Pi* Pisz, *Pl* Płock, *Po* Poznań, *Rz* Rzeszów, *Sk* Skierniewice, *Sw* Świdnica, *Wa* Warszawa, *Wr* Wrocław, *Zi* Zielona Góra; the city abbreviation is followed by the centre number ^b^ Shadowed boxes indicate presumable associations among isolates; ^c^ *rep1, rep2, rep17, rep18* – plasmid replicon families according to Jensen et al., 2010 [24]; TA1, TA2- *axe-txe* and ω*-ε-ζ* stabilization systems specific for pRUM and pRE25, respectively; ^d^ Presumably C1 transposon type, however no amplification of the region containing IS*1251* could be obtained, in spite of several attempts

^e^Results from Wardal et al., 2014 [18]

## Discussion

Currently, VR*Efm* play an increasingly important role in nosocomial infections and are considered alert pathogens [29], with *vanA* as a main determinant of this phenotype within many countries [30]. In Poland VRE remain less prevalent than in the United States or some European countries, e.g. our recent study revealed 7 % vancomycin-resistance among invasive *E. faecium* collected during 2010–2011 [31]. Although we observed an increasing prevalence of VanB *E. faecium* [17], VanA is still most frequent among Polish VR*Efm* ([31] and NRCST unpublished observations). In the present study we aimed at the characterization of clonality of VanA*-*VR*Efm* and genetic elements associated with this resistance determinant. Numerous reports show that in the case of human nosocomial infections vancomycin resistance is almost exclusively acquired by the hospital-adapted meroclone of *E. faecium*, now widespread all over the world [2, 3] and prevalent among invasive *E. faecium* in Polish hospitals [31]. In this study, the *vanA* determinant was carried by representatives of this meroclone with only a few exceptions limited to the 1997–2005 period. These isolates might represent intermediates, by which glycopeptides resistance determinants were introduced into hospitals. All isolates belonging to hospital meroclone, as expected, were resistant to both ampicillin and ciprofloxacin, and enriched in putative virulence traits / markers such as IS*16*, *esp*_Efm_, *intA*_Efm_ and pili genes. The population structure determined for Polish VR*Efm* VanA closely resembled these of hospital-associated *E. faecium* in other countries. High diversity of STs/MTs is consistent with the presence of polyclonal hospital population of *E. faecium* that subsequently acquires vancomycin resistance determinants [13, 14]. The vast majority of isolates grouped into hospital lineage 17/18, mostly represented by STs 17, 117, 18, 132, 202 and lineage 78, which included STs 78, 192 and 412. In contrast to several other countries, where ST203 and ST16 constituted a significant proportion of hospital *E. faecium* [3, 14, 32, 33], in our population only one representative of ST16 was found and ST203 was completely absent. The characteristic change in the proportion of isolates belonging to lineage 17/18 and lineage 78 was observed since the year 2005 when lineage 78 started to be significantly more frequent in Poland. Our results are in agreement with observations made in other studies, suggesting waves of successful *E. faecium*, first from lineage 17/18 and followed by lineage 78 strains [4, 34]. This population shift, apparent in MLST and MLVA, was additionally associated with a change in plasmidome composition and observed decreased resistance levels to tetracycline and aminoglycosides.

Diversity of Tn*1546* in VR*Efm* is typical for this transposon, as reported by others [13, 14]. Nevertheless, in the present study we observed several new variants of Tn*1546*. VanA transposons indistinguishable from the Tn*1546* A1 prototype [12] were frequently encountered in Europe, especially in the late 1990s and 2000s [14, 27]. This type and its mutational derivatives (A2-A6) were ubiquitous among early VR*Efm* in our study. Single-nucleotide T insertion between nt 9063–9064 in the *vanY* gene of B4 type of transposon resulted in a translational frameshift and a truncated translation product. This change, however, did not abolish the glycopeptide resistance. VanY is a membrane-associated D,D-carboxypeptidase that hydrolyses the C-terminal D-Ala or D-Lac residue of peptidoglycan precursors but lacks transpeptidase activity. VanY, together with VanZ, represent accessory proteins, which are not required for the expression of glycopeptide resistance but increase its level [35]. Isolates with a deletion of *vanY* gene showed lower resistance levels to teicoplanin, likely due to the diminished transcription of *vanZ* while point mutations in *vanY*, observed so far were not associated with a loss of protein function [27, 36].

**Fig. 3:**
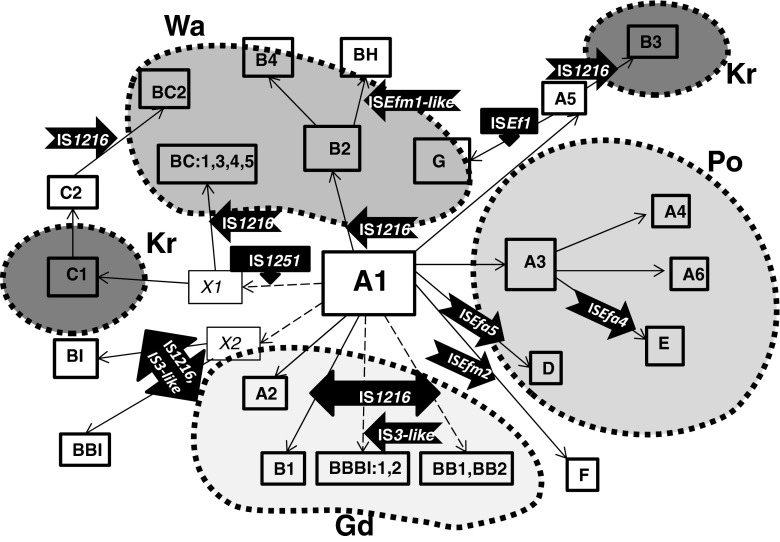
Hypothetical evolution of Tn*1546* structures among Polish VR*Efm* VanA. Type A1, found in different cities, is a presumable ancestor variant with remaining types being its direct or indirect derivatives. Types A2, A3 and A5 developed by point mutations in *wt* type. A4 developed from A3 through single deletion events in the *vanS-vanH* intergenic region. A6 is an A3 derivative that lacks ca. 1900 bps in the 5′ end. The E type transposon, a third potential derivative of A3, arose through acquisition of IS*Efa4* between *vanX* and *vanY*. A3 and its derivatives were typical for Poznań (Po) medical centres. B3 and G variants presumably developed from A5 after insertion events of IS*1216* and IS*Ef* within *vanX-vanY* intergenic region in Kraków (Kr) and Warsaw (Wa), respectively. The ubiquitous B2 type, typical for Warsaw, probably emerged from a single insertion event of IS*1216* within the A1 type with a concomitant deletion of the 5′ end of the transposon. The B4 (additional single nucleotide insertion within *vanY*) and BH (IS*Efm1*-like insertion between *vanX* and *vanY*) types represent possible derivatives of B2. The B1, D and F transposon types are potential derivatives of A1 formed by IS*1216*, IS*Efa5* and IS*Efm2* insertions, respectively. Another group of transposon variants, encompassing types BI, BBI, BBBI1, BBBI2, BB1 and BB2 emerged through complex insertion and deletion events in different regions of *wt* transposon promoted mostly by IS*1216* elements. This group was detected mainly in Gdańsk (Gd). The activity of another insertion sequence, IS*1251*, followed by IS*1216* insertions and several point mutations resulted in the formation of C- and BC-types in Kraków and Warsaw, respectively

Activity of various ISs represented a very important factor, contributing to the formation of several novel transposon types. IS*1216*, the most common IS in our study, characteristic for B-types, was detected at various positions of the transposon, and its insertion often resulted in deletions of adjacent sequences in ORF2, *vanX* and the *vanX-vanY* intergenic region, as observed by others [13, 27]. The BI, BBI, BBBI types, apart from IS*1216*, exhibited the concomitant presence of a IS*1216V*-IS*3*-like element, originally reported in 1995 [37]. Since then, this element was described in several studies, which reported intact as well as a 5′-truncated IS*3*-like sequence [27, 38], both of which were also detected in our study. The C-type, harbouring IS*1251*, was relatively frequent and the integration sites of this IS were identical to those published by others [14, 37, 39]. Sequencing analysis allowed discerning the C1-type, specific for Krakow hospitals and the C2-type, found in other cities. The D type transposon, containing IS*Efa5*, to our knowledge, represents the first example of this variant outside of South America [34]. Type E represents the first insertion of IS*Efa4* in the *vanX-vanY* intergenic region. This IS was described earlier in *orf2*-*vanR* and *vanS*-*vanR* intergenic regions [40, 41], as well as within IS*1542* [42]. The F type harboured IS*Efm2*, representative of the IS*256* family. Thus far, in enterococci this IS has been solely observed inserted between *orf13* and *tetS* within CTn*6000* transposon [43]. In our study, we report for the first time the insertion of IS*Efm2* into Tn*1546*. The G type transposon with IS*Ef1*, reported earlier for the VR*Efm* outbreak in two neighbouring Warsaw medical centres in 2009 [18] was additionally detected in isolates from 2009 to 2010 derived from seven different cities, which may indicate its multicenter spread. Integration of the IS*Efm1*-like element, belonging to the IS*982* family in the BH type, represents yet another novel insertion event in Tn*1546*. The presence of IS*Efm1* was described previously in the *vanX-vanY* intergenic region [44], as well as within the *vanD* operon [45]. Complex analysis of transposon structures described in our study revealed the potential scheme of their hypothetical evolution among Polish VR*Efm*. In this scenario, we propose the A1 type as presumable ancestor variant, with remaining types being its direct or indirect derivatives (Fig. 3).

The *vanA* determinants are almost exclusively located on plasmids and these elements play a very important role in the spread of glycopeptides resistance [46]. Our results show that the Polish VR*Efm* population is enriched in plasmid replicons of different families including megaplasmids, Inc18-, pRUM- and pMG1/pHT-like plasmids, encountered in VanA-VR*Efm* in other countries [16, 46, 47]. Size variation of *vanA-*plasmids, even within the same family indicates their flexibility, and identification of multiple *rep* types in a single plasmid suggests a common presence of plasmid cointegrates. We observed an interesting change in the VanA-associated plasmidome between early (1997–2005) and more recent (2006–2010) isolates. In particular, *rep1-vanA* replicons were quite abundant among early isolates and typically located on plasmids over 140 bp in size. Together with *rep2-vanA* replicons they represent the Inc18 family, associated with Tn*1546* elements among clinical *E. faecium* in Europe [16, 47]. Another shift in plasmidome composition between early and recent isolates was shown for pMG1 replicons, present exclusively among early VR*Efm*. High prevalence of pMG1-like elements was observed among VR*Efm* in the United States and Japan, where they contributed to the spread of both aminoglycoside and glycopeptide resistance [46]. Apart from Inc18 plasmids, the pRUM derivatives constitute the second main carrier of vancomycin resistance among the contemporary *E. faecium* isolates [16, 46]. Plasmids with the pRUM-like *rep* can be divided into two groups, one with *axe-txe* genes and *mob* regions from the staphylococcal pC223 plasmid and the other with relaxase from pEF1 and lacking *axe-txe* [15, 16, 46]. Our results indicate that representatives of both these groups are present among Polish VR*Efm*. Additionally, we observed plasmids with unknown *rep* types among isolates obtained since 2006, which suggests the appearance of a new *vanA-*plasmid type(s), not included in the available classification scheme [24] and which will be a subject to further studies.

Finally, the analysis of PFGE-S1 hybridization results in the context of epidemiological information, determined Tn*1546* types and the clonal background of the isolates, which revealed a high complexity of genetic events involving VR*Efm* with VanA phenotype and resulting in the dissemination of this type of resistance. As these 52 isolates were pre-selected for a maximal representation of the collection diversity, only a few examples of clonal spread were observed, such as dissemination of ST132 strain with a 45-kb plasmid harbouring C1 transposon in a Krakow hospital (Table 2). The role of VR*Efm* clonal spread in Poland, however, had been demonstrated before in our outbreak studies [18, 39, 48]. Particular types of transposons in the analysed group were frequently associated with various plasmid vectors. This situation may have resulted from transposition of Tn*1546* among plasmids [12], promoted by integration ‘hot-spots’ [49] and from the recombination processes among enterococcal plasmids [15, 50]. The present study also provided examples of involvement of plasmids as vectors of vancomycin resistance, by demonstrating the presence of plasmids of the same size and with the same transposon types and plasmid-specific genes in different strains, as found in other studies [15, 51]. Occasionally, *vanA-*plasmids appeared to be integrated into bacterial chromosome, in agreement with other observations [52]. Further detailed studies employing extensive sequencing are indispensable to fully elucidate the events involving genetic elements engaged in the dissemination of the *vanA* gene cluster in the population of Polish VR*Efm*.

In conclusion, the VR*Efm* of the VanA phenotype collected in our country over the period 1997–2010 represent a highly variable group in the respect of their clonal composition, plasmid content and structures of Tn*1546*, a direct carrier of *vanA* genes. High genetic plasticity of these organisms, together with a rapid global spread of successful hospital-adapted enterococcal clones constitute a significant and continuously increasing epidemiological threat for human health. Thus, both epidemiological situation concerning VR*Efm* as well as genetic elements and strains associated with VanA vancomycin resistance warrant further studies.

## Electronic supplementary material

Below is the link to the electronic supplementary material.

ESM 1 (DOCX 20 kb)

ESM 2 (DOCX 58 kb)

ESM 3 (DOCX 16 kb)
