# Supplementary material for: Diversity of plasmids and Tn1546-type transposons among VanA Enterococcus faecium in Poland
Source: Eur J Clin Microbiol Infect Dis. 2016 Oct 17;36(2):313–28. doi: 10.1007/s10096-016-2804-8 (PMC5253160; doi:10.1007/s10096-016-2804-8)
Supplement: Supplementary file 1 — (DOCX 20 kb) [file 10096_2016_2804_MOESM1_ESM.docx]

**Supplementary Table 1.** Primers used for Tn*1546* typing and detection of IS*16, esp*_Efm,_ *fms21 (pilA), rep*_pLG1,_ plasmid addiction systems, relaxase genes and *intA*_ICE_*_Efm1_*.

| **Tn*1546* typing and detection of epidemiological markers** | | | | | | | |
| --- | --- | --- | --- | --- | --- | --- | --- |
| Primer names | Sequence (5’-3’) | | Position in reference sequence | | Reference sequence | | Literature |
| tn1546-1 | GGAAAATGCGGATTTACAACGCTAAG | | 13-38 | | M97297 | | ^1^ |
| ORF1_3 | AAAAGGAGCCACCATCTACCG | | 921-901 | | M97297 | | ^2^ |
| ORF1_5 | CACGTCCTGCCGACTATGATTATTT | | 1915-1891 | | M97297 | | ^2^ |
| ORF2_F | TCATTCCATTTCTGTATTTTCAATTT | | 3048-3073 | | M97297 | | ^3^ |
| ORF2_R | GCCCATTAGCGGAATACAGA | | 3786-3767 | | M97297 | | ^3^ |
| ORF2_F2 | ACTAATGTATCTAGGGCTTCA | | 3709-3729 | | M97297 | | ^3^ |
| vanR_R | GCAATTTCATGTTCATCATCCA | | 4019-3998 | | M97297 | | ^3^ |
| vanS | AACGACTATTCCAAACTAGAA | | 4676-4696 | | M97297 | | ^2^ |
| vanH2 | GAGCATGGAATGCATCTGCC | | 6081-6062 | | M97297 | | ^4^ |
| vanS1 | ATTGTTCAGCATGGAGGGC | | 5696-5714 | | M97297 | | ^4^ |
| vanA1 | CATGAATAGAATAAAAGTTGCAATA | | 6978-7002 | | M97297 | | ^5^ |
| vanA2 | CCCCTTTAACGCTAATACGATCAA | | 8007-7984 | | M97297 | | ^5^ |
| vanX2 | TTATTTAACGGGGAAATC | | 8624-8607 | | M97297 | | ^6^ |
| vanX1 | ATGGAAATAGGATTTACTT | | 8016-8034 | | M97297 | | ^6^ |
| vanY1 | AGAGACGAACCATACCCCAA | | 8577-8596 | | M97297 | | ^3^ |
| vanX_F | ATGGGTATTTTCAGAAGTCCC | | 9213-9193 | | M97297 | | ^3^ |
| vanY2-R | AGTATGTGTTGATCCGGGAAAC | | 9948-9927 | | M97297 | | ^7^ |
| vanZ1 | CTGGGAATTTCAGAGAGATG | | 10258-10277 | | M97297 | | ^4^ |
| vanZ2 | AATGGGTACGGTAAACGAGC | | 10581-10562 | | M97297 | | ^4^ |
| tn1546-2 | GGAAAATGCGGATTTACAACGTTAAG | | 10839-10814 | | M97297 | | ^1^ |
| IS1216F | CCGTGGGCTACTATCTTCGTT | | 124-144 | | U49512 | | ^8^ |
| IS1216R | AATTTATTGCGTCTCTTTACTGGA | | 610-587 | | U49512 | | ^8^ |
| IS1216-IS3-like | CTACAGACGGGTTACGGTT | | 858-876 | | AY916786 | | this study |
| IS16F | CATGTTCCACGAACCAGAG | | 24327-24346 | | AF507977 | | ^9^ |
| IS16R | TCAAAAAGTGGGCTTGGC | | 24873-24855 | | AF507977 | | ^9^ |
| intA-up | GAGCCAATTCAGGATGTCG | | 192–210 | | intA /ABQJ01000139 | | ^10^ |
| intA-dn | GAAAGAAGGATACGGGAAGGT | | 669–649 | | intA /ABQJ01000139 | | ^10^ |
| ESP 14F | AGATTTCATCTTTGATTCTTGG | | 1341-1362 | | AF034779 | | ^11^ |
| ESP 12R | AATTGATTCTTTAGCATCTGG | | 1851-1831 | | AF034779 | | ^11^ |
| fms21F | CTTATTGGAATGTTAGGAATCAT | | 1184-1203 | | fms21(pilA)/TX0016 | | ^12^ |
| fms21R | TCAGTAGCAGTCAGCTTTCC | | 1941-1919 | | fms21(pilA)/TX0016 | | ^12^ |
| **Plasmid-associated genes** | | | | | | | |
| Primer names | | Sequence (5’-3’) | | Length of amplified PCR fragment in bp | | Literature | |
| rel-pRE25-1 | | AATGGCTTCTTATCGAAGTGG | | 325 | | ^13^ | |
| rel-pRE25-2 | | TTCATCATCTCTATGAATGG | |  |  |  |  |
| rel-pCIZ2-1 | | ATGGCAACAGTTAAAGTAAG | | 208 | | ^13^ | |
| rel-pCIZ2-2 | | CAGCGGTAATTTCTCCTGGTC | |  |  |  |  |
| rel-pRUM-1 | | AATGTATGGCAAAACAGATGG | | 138 | | ^13^ | |
| rel-pRUM-2 | | ACGGCAACTTGATGATTAGG | |  |  |  |  |
| rel-pEF1-1 | | AAGCAAATCTTCCAAAGAGG | | 217 | | ^13^ | |
| rel-pEF1-2 | | GGTGAATCGTTGCCATCCAGTAGCC | |  |  |  |  |
| rel-pHTb-1 | | TAAAGATTTGCGTGAAATGG | | 192 | | ^13^ | |
| rel-pHTb-2 | | TCTTGGAACATCACCGATCC | |  |  |  |  |
| relBE-F | | CAGAGAATGCGTTTGACCG | | 456 | | ^14^ | |
| relBE-R | | GGTGTAACTCCTTCTGAAGCG | |  |  |  |  |
| mazEF1-F | | CTTCGTTGCTCCTCTTGC | | 496 | | ^14^ | |
| mazEF1-R | | CGTTGGGGAAATTCACCG | |  |  |  |  |
| axe-txe-F | | CTGACCCTTTCCTTACTTCCG | | 556 | | ^14^ | |
| axe-txe-R | | GGGTGAAAGGAATGGAAGCAG | |  |  |  |  |
| e-z-F | | GTGGTTTAGGTGGCTGCAAG | | 1044 | | ^14^ | |
| e-z-R | | TTAACGAATTATCGGCAAGC | |  |  |  |  |
| efm-repA_pLG1-up | | GAAAATGATATCTACTTACTCG | | 568 | | ^7^ | |
| efm-repA_pLG1-dn | | TTACATAGACAAAAATCAGGT | |  |  |  |  |

1. Palepou MF, Adebiyi AM, Tremlett CH et al. Molecular analysis of diverse elements mediating VanA glycopeptide resistance in enterococci. *J Antimicrob Chemother* 1998; **42**: 605-12.

2. Huh JY, Lee WG, Lee K et al. Distribution of insertion sequences associated with Tn*1546*-Like elements among *Enterococcus faecium* isolates from patients in Korea. *J Clin Microbiol* 2004; **42**: 1897-902.

3. Talebi M, Pourshafie MR, Katouli M et al. Molecular structure and transferability of Tn*1546*-like elements in *Enterococcus faecium* isolates from clinical, sewage, and surface water samples in Iran. *Appl Environ Microbiol* 2008; **74**: 1350-6.

4. Jensen LB, Ahrens P, Dons L et al. Molecular analysis of Tn*1546* in *Enterococcus faecium* isolated from animals and humans. *J Clin Microbiol* 1998; **36**: 437-42.

5. Clark NC, Cooksey RC, Hill BC et al. Characterization of glycopeptide-resistant enterococci from U.S. hospitals. *Antimicrob Agents Chemother* 1993; **37**: 2311-7.

6. Yu HS, Seol SY, Cho DT. Diversity of Tn*1546*-like elements in vancomycin-resistant enterococci isolated from humans and poultry in Korea. *J Clin Microbiol* 2003; **41**: 2641-3.

7. Wardal E, Markowska K, Zabicka D et al. Molecular analysis of VanA outbreak of *Enterococcus faecium* in two Warsaw hospitals: the importance of mobile genetic elements. *Biomed Res Int* 2014: 575367.

8. Tsai JC, Hsueh PR, Chen HJ et al. The *erm(T)* gene is flanked by IS*1216V* in inducible erythromycin-resistant *Streptococcus gallolyticus* subsp. *pasteurianus*. *Antimicrob Agents Chemother* 2005; **49**: 4347-50.

9. Werner G, Fleige C, Geringer U et al. IS element *IS16* as a molecular screening tool to identify hospital-associated strains of *Enterococcus faecium*. *BMC Infect Dis* 2011; **11**: 80.

10. Sadowy E, Sienko A, Gawryszewska I et al. High abundance and diversity of antimicrobial resistance determinants among early vancomycin-resistant *Enterococcus faecium* in Poland. *Eur J Clin Microbiol Infect Dis* 2013; **32**: 1193-203.

11. Vankerckhoven V, Van Autgaerden T, Vael C et al. Development of a multiplex PCR for the detection of *asa1*, *gelE*, *cylA*, *esp*, and *hyl* genes in enterococci and survey for virulence determinants among European hospital isolates of *Enterococcus faecium*. *J Clin Microbiol* 2004; **42**: 4473-9.

12. Sillanpaa J, Nallapareddy SR, Prakash VP et al. Identification and phenotypic characterization of a second collagen adhesin, Scm, and genome-based identification and analysis of 13 other predicted MSCRAMMs, including four distinct pilus loci, in *Enterococcus faecium*. *Microbiology* 2008; **154**: 3199-211.

13. Freitas AR. Ecology and evolution of antimicrobial resistance in *Enterococcus*: A multilayered molecular approach with emphasis in plasmid diversity. *PhD Thesis* 2011.

14. Moritz EM, Hergenrother PJ. Toxin-antitoxin systems are ubiquitous and plasmid-encoded in vancomycin-resistant enterococci. *Proc Natl Acad Sci U S A* 2007; **104**: 311-6.
