# Supplementary material for: Diversity of plasmids and Tn1546-type transposons among VanA Enterococcus faecium in Poland
Source: Eur J Clin Microbiol Infect Dis. 2016 Oct 17;36(2):313–28. doi: 10.1007/s10096-016-2804-8 (PMC5253160; doi:10.1007/s10096-016-2804-8)
Supplement: Supplementary file 2 — (DOCX 58 kb) [file 10096_2016_2804_MOESM2_ESM.docx]

**Supplementary Table 2.** STs and MTs among VR*Efm* VanA isolates in Poland, 1997-2010.

| **ST^a^** | **MT^a^** | **Centre** | **Year of isolation** |
| --- | --- | --- | --- |
| **Lineage 17/18** | | | |
| 16 | 249 | Rz | 2006 |
| 17 (28) | 1 (14), 4 (2), 11 (4), 294, 296, 340, nt (5) | By-2, Gdy, Gr, In, Ka, Ke, Kr-1, Pi, Po-1, Po-2, Po-3, Po-5, Wa-1, Wa-4, Wa-10 | 2001, 2002, 2003, 2005, 2006, 2008, 2009, 2010 |
| 18 (22) | 1 (10), 3 (2), 7 (5), 144, 324 (4) | Gd, Ko, Kr-1, Kr-4, Op, Osw, Po-4, Wa-4, Wa-10 | 1998, 2003, 2004, 2005, 2008, 2009, 2010 |
| 19 | 329 | Po-2 | 2003 |
| 64 (2) | 1, 324 | Gd, Waw-1 | 1998, 2007 |
| 80 (2) | 5, 7 | Waw-2 | 2009, 2010 |
| 117 (27) | 12 (3), 25 (22), 127, 273 | K1, Po-2, Wa-6 | 2002, 2003, 2004, 2005, 2010 |
| 125 | 10 | Gd | 1999 |
| 132 (19) | 13 | Kr-1, Kr-2, Mi | 2003 |
| 202 (12) | 1 (4), 4 (4), 7 (3), 11 | Gr, Ka, Kr-1, Po-1, Po-2, Wa-1, Wa-7, Wa-10 | 2002, 2003, 2007, 2008, 2009, 2010 |
| 210 | 323 | Gda | 1997 |
| 262 (2) | 10 | Wa-4 | 2009 |
| 279 (8) | 1 (4), 231 (4) | Po-2, Kr-1, Lo, Sk, Zi | 2002, 2003, 2005, 2006, 2010 |
| 381 | 264 | Gd | 1999 |
| 382 (7) | 325 (2), 327 (2), nt (3) | By-1, Po-1, Po-2 | 2002, 2005 |
| 384 | nt | Po-1 | 2002 |
| 385 (5) | 295 (2), 300, 328, 372 | Po-1, Po-2, Po-4 | 2001, 2002 |
| 386 (4) | 4 (1), 294 (2), 330 | Kr-1, Po-1, Po-3 | 2001, 2002, 2003 |
| 387 (3) | 50 (3) | Kr-1, Kr-3 | 2003 |
| 388 | 1 | Kr-1 | 2005 |
| 389 | 331 | Kr-1 | 2005 |
| 407 | 1 | Gd | 1997 |
| 408 | 12 | Gd | 1997 |
| 409 | 13 | Kr-1 | 2003 |
| 410 (3) | 325 (2) | Po-2 | 2003 |
| 561 | 12 | Wa-2 | 2010 |
| 563 (3) | 2005 (3) | Kr-2, Po-2 | 2004, 2005 |
| 780 (3) | 12 (3) | Gr, Wa-3, Wa-5 | 2010 |
| 877 | 12 | Sw | 2010 |
| 920 | 1 | Po-5 | 2006 |
| 975 | 12 | Po-1 | 2002 |
|  |  |  |  |
| **Lineage 78** | | | |
| 78 (21) | 12 (2),25, 159 (17), 334 | Gr, Kos, Os, Ost, Ot, Wa-1, Wa-2, Wa-4, Wa-10, Wr | 2006, 2007, 2008, 2009, 2010 |
| 192 (9) | 12 (2), 50, 159 (5) | Poz-1, Poz-2, Wa-1, Wa-8, Wa-10, | 2002, 2003, 2007, 2008, 2009 |
| 341 (2) | 159 (2) | Kr-5, Wa-2 | 2010 |
| 412 (9) | 159 (9) | Wa-2, Wa-9 | 2007, 2010 |
| 549 | 159 | Po-4 | 2007 |
|  | | | |
| **CC22** | | | |
| 56 (1) | 302 | Po-2 | 2003 |
|  | | | |
| **Singletons** | | | |
| 162 (2) | 373 (2) | Pl, Pt | 2004 |
| 265 | 314 | Kr-1 | 2005 |
| 411 (5) | 27 (5) | Gd | 1998 |

Medical centres are given in abbreviations: *By,* Bydgoszcz; *Gd-a* Gdańsk, adult haematology ward; *Gd-p,* Gdańsk, paediatric haematology ward; *Gdy,* Gdynia; *Gr,* Grodzisk Mazowiecki; *In,* Inowrocław; *Ka,* Katowice; *Ke,* Kętrzyn; *Ko,* Konin; *Kos,* Kościerzyna; *Kr,* Kraków; *Lo,* Łódź; *Mi,* Mielec; *Op,* Opole; *Os,* Ostrów Mazowiecki; *Osw,* Ostrów Wielkopolski; *Ost,* Ostrzeszów; *Ot,* Otwock; *Pi,* Pisz; *Pl,* Płock; *Po,* Poznań; *Rz,* Rzeszów; *Sk,* Skierniewice; *Sw,* Świdnica; *Wa,* Warszawa; *Wr,* Wrocław; *Zi,* Zielona Góra; the city abbreviation is followed by the centre number; ^a^ number of isolates with particular MT/ST, if different from one, are given in brackets
