# Supplementary material for: Diversity of plasmids and Tn1546-type transposons among VanA Enterococcus faecium in Poland
Source: Eur J Clin Microbiol Infect Dis. 2016 Oct 17;36(2):313–28. doi: 10.1007/s10096-016-2804-8 (PMC5253160; doi:10.1007/s10096-016-2804-8)
Supplement: Supplementary file 3 — (DOCX 16 kb) [file 10096_2016_2804_MOESM3_ESM.docx]

**Supplementary Table 3.** Tn*1546* transposon types distribution in VanA *E. faecium* according to MLST types, medical centres and the year of isolation.

| **Tn*1546* type** | **No. of isolates** | **Medical Centre^a^** | **MLST^a^** | **Year of isolation^a^** |
| --- | --- | --- | --- | --- |
| A1 | 13^b^ | Gd(6), Po-3(2), Pl, Ot, Ka, Wa-4, Wa-10 | 411(4), 18(3), 17(2), 162(2), 78, 386 | 1998(6), 2004(2), 2009(2), 2003(1),2008 |
| A2 | 1 | Gd | 411 | 1998 |
| A3 | 36 | Po-2(24), Po-1(11), Po-4 | 117(13), 382(5), 202(4), 410(3), 17(2), 385(2), 386(2), 19, 56, 192, 279, 384 | 2002(19), 2003(11), 2001(2), 2004(2), 2005(2) |
| A4 | 2 | Po-1(2) | 385(2) | 2001(2) |
| A5 | 1 | Gr | 78 | 2010 |
| A6 | 1 | Po-4 | 549 | 2007 |
| B1 | 3 | Gd (3) | 18(2), 64 | 1998(3) |
| B2 | 38 | Wa-1(11), Wa-2(8), Os(2), Po-5(2), Wr(2), Gr, Ke, Op, Osw, Pi, Sk, Sw, Wa-3, Wa-5, Wa-6, Wa-7, Wa-8, Wa-9 | 117, 17(3), 18(2), 192(3), 202(2), 279, 341, 412(3), 561, 64, 78(13), 780(3), 80(2), 877, 920 | 2010 (17), 2007(8), 2006(5), 2008(5), 2009(3) |
| B3 | 1 | Kr-5 | 341 | 2010 |
| B4 | 2 | Wa-1, By-1 | 78, 382 | 2005(2) |
| C1 | 40 | Kr-1(36), Kr-2(2), Kr-3, Kr-4 | 132(18), 17(7), 18(4), 387(3), 117, 202, 279(2), 386, 388, 389, 409 | 2003(30), 2005(9), 2004 |
| C2 | 5 | Gdy(3), Wa-4, Rz | 17(4), 16 | 2010(3), 2005, 2006 |
| D | 7 | Po-1(5), Po-2(2) | 17(4), 192(2), 382 | 2002(6), 2001 |
| E | 14 | Po-2(14) | 117(12), 563(2) | 2003(5), 2004(5), 2005(4) |
| F | 1 | Lo | 279 | 2005 |
| G | 25^b^ | Wa-10(11), Wa-4(7), Ko, Ost, Gr, Ot, In, By-2, Po-4 | 18(11), 17(5), 78(3), 192(2), 202(2), 262(2) | 2009(22), 2010(3) |
| BB1 | 1 | Gd | 210 | 1998 |
| BB2 | 1 | Gd | 381 | 1999 |
| BC1 | 2 | Wa-2(2) | 412(2) | 2007(2) |
| BC2 | 3 | Wa-1(3) | 17, 18, 202 | 2008(3) |
| BC3 | 1 | Wa-2 | 412 | 2010 |
| BC4 | 1 | Wa-2 | 412 | 2010 |
| BC5 | 2 | Wa-2(2) | 412(2) | 2010(2) |
| BH | 1 | Gr | 202 | 2008 |
| BI | 1 | Kos | 78 | 2008 |
| BBI | 1 | Ka | 202 | 2010 |
| BBBI1 | 2 | Gd | 125, 407 | 1997, 1999 |
| BBBI2 | 1 | Gd | 408 | 1997 |
| untypeable | 9 | Po-1 (2), Po-2 (1), Mi (1), Kr-1 (1), Kr-2 (1), Zi (3) | 132 (1), 192 (1), 265(1), 279 (3), 385 (1), 563 (1), 975 (1) | 2002(3), 2003(1), 2004(1), 2005(1), 2010(3) |

Medical centres are given in abbreviations: *By,* Bydgoszcz; *Gd,* Gdańsk; *Gdy,* Gdynia; *Gr,* Grodzisk Mazowiecki; *In,* Inowrocław; *Ka,* Katowice; *Ke,* Kętrzyn; *Ko,* Konin; *Kos,* Kościerzyna; *Kr,* Kraków; *Lo,* Łódź; *Mi,* Mielec; *Op,* Opole; *Os,* Ostrów Mazowiecki; *Osw,* Ostrów Wielkopolski; *Ost,* Ostrzeszów; *Ot,* Otwock; *Pi,* Pisz; *Pl,* Płock; *Po,* Poznań; *Rz,* Rzeszów; *Sk,* Skierniewice; *Sw,* Świdnica; *Wa,* Warszawa; *Wr,* Wrocław; *Zi,* Zielona Góra; ^a^ number of isolates, if different from one, are given in brackets. ^b^ 2 and 18 izolates representing types A1 and G , respectively, were published elsewhere [1].

1. Wardal E, Markowska K, Zabicka D et al. Molecular analysis of *vanA* outbreak of *Enterococcus faecium* in two Warsaw hospitals: the importance of mobile genetic elements. *Biomed Res Int* 2014: 575367.
